# Exploring potential genes and mechanisms linking erectile dysfunction and depression

**DOI:** 10.3389/fendo.2023.1221043

**Published:** 2023-12-04

**Authors:** Penghui Yuan, Yinwei Chen, Taotao Sun, Lingang Cui, Yinsheng Wei, Teng Li, Qingjun Meng

**Affiliations:** ^1^ Department of Urology, The First Affiliated Hospital of Zhengzhou University, Zhengzhou, China; ^2^ Department of Urology Tongji Hospital, Tongji Medical College, Huazhong University of Science and Technology, Wuhan, Hubei, China; ^3^ Reproductive Medicine Center, Tongji Hospital, Tongji Medical College, Huazhong University of Science and Technology, Wuhan, Hubei, China

**Keywords:** erectile dysfunction, depression, biomarker, shared transcriptome, bioinformatics

## Abstract

**Background:**

The clinical correlation between erectile dysfunction (ED) and depression has been revealed in cumulative studies. However, the evidence of shared mechanisms between them was insufficient. This study aimed to explore common transcriptomic alterations associated with ED and depression.

**Materials and methods:**

The gene sets associated with ED and depression were collected from the Gene Expression Omnibus (GEO) database. Comparative analysis was conducted to obtain common genes. Using R software and other appropriate tools, we conducted a range of analyses, including function enrichment, interactive network creation, gene cluster analysis, and transcriptional and post-transcriptional signature profiling. Candidate hub crosslinks between ED and depression were selected after external validation and molecular experiments. Furthermore, subpopulation location and disease association of hub genes were explored.

**Results:**

A total of 85 common genes were identified between ED and depression. These genes strongly correlate with cell adhesion, redox homeostasis, reactive oxygen species metabolic process, and neuronal cell body. An interactive network consisting of 80 proteins and 216 interactions was thereby developed. Analysis of the proteomic signature of common genes highlighted eight major shared genes: CLDN5, COL7A1, LDHA, MAP2K2, RETSAT, SEMA3A, TAGLN, and TBC1D1. These genes were involved in blood vessel morphogenesis and muscle cell activity. A subsequent transcription factor (TF)–miRNA network showed 47 TFs and 88 miRNAs relevant to shared genes. Finally, CLDN5 and TBC1D1 were well-validated and identified as the hub crosslinks between ED and depression. These genes had specific subpopulation locations in the corpus cavernosum and brain tissue, respectively.

**Conclusion:**

Our study is the first to investigate common transcriptomic alterations and the shared biological roles of ED and depression. The findings of this study provide insights into the referential molecular mechanisms underlying the co-existence between depression and ED.

## 1 Introduction

Erectile dysfunction (ED) is a common male sexual dysfunction that increases with age (1), and it could have a negative effect on both physical and psychological health for couples. Data reported that 322 million men would suffer from ED to varying degrees by 2025 (2). There are a multitude of factors that contribute to the pathogenesis of ED, including neurogenic, vascular, endocrinological, and psychological factors. Therefore, it is believed that ED is strongly associated with these diseases. For example, ED could be an early predictor of adverse cardiovascular events (3, 4). Understanding the crosslinks between ED and related diseases is vital for their treatment.

Psychological factors are involved in the process of ED. Therefore, ED may be associated with psychological illness. Depression is a common mental illness regarded as one of the most severe public concerns (5). As a result, major depressive disorder has been deemed the most prevalent mental disorder in the world (6). The interactive relationship between ED and depression has been explored in cumulative studies. Depression occurred in 8.7%–43.1% of patients in ED (7, 8). Liu et al. (3) reported that men with depression were 1.39 times more likely to get impotence. Sexual dysfunction was significantly correlated with the severity of depression (9). Furthermore, ED was 2.92 times more likely to lead to depression. Nocturnal penile tumescence time and rigidity were impaired in depressed men. Preliminarily, biological factors have been proposed as contributing to the co-occurrence of these conditions, except for psychological factors. Goldstein et al. (10) postulated that the hypothalamic–pituitary–adrenocortical axis affected by depression could produce excessive catecholamine, impairing cavernosal muscle relaxation and propelling ED. Moreover, decreased testosterone level has been established as a risk factor for ED (11). Chou et al. (12) found that patients with depression had a lower level of testosterone compared with controls, and testosterone therapy showed a promising effect on depressive symptoms. Furthermore, a significant association between sexual dysfunction including ED and the dosage of antidepressants as well as benzodiazepines was noted (13). Furthermore, men with ED treated with phosphodiesterase-5 inhibitors or penile prostheses had lower depression rates than those who did not receive treatment (14). ED and depression are likely caused by multidimensional mechanisms, and evidence from current studies is insufficient to explain their shared pathogenesis.

Microarray and high-throughput sequencing technologies have advanced pathogenetic research in recent years (15). Mining the common alternations at the molecular level will broaden our understanding of potential mechanisms in ED and depression. Therefore, the study aimed to explore shared transcriptomic profiles between ED and depression. In the present article, we have explored the transcriptome specific to ED and depression using tissue and human samples for the first time. Associated biological markers and signaling pathways were identified and validated adequately. This study would contribute to the theoretical research on coexisting ED and depression as well as promising therapeutic guidance for future studies.

## 2 Materials and methods

### 2.1 Data source

The transcriptomic data associated with ED and depression were collected from the Gene Expression Omnibus (GEO) database (https://www.ncbi.nlm.nih.gov/geo/) based on the diagnostic criteria of the diseases in December 2022 (1, 16). The present study focused on major depressive disorder, one of the most prevalent mental disorders worldwide. GSE2457, a dataset of ED, deposited expression profiling of corpus cavernosum by array from 10 diabetes-induced ED in rats and controls (17). GSE54564, a dataset of depression, contained expression data of the human brain amygdala from 42 patients with major depressive disorders and non-psychiatric control subjects pair-matched by age and ethnicity (18, 19). In datasets for validation, GSE206528 incorporated single-cell transcriptome of the corpus cavernosum from eight patients with ED and controls (20). Genes related to depression were retrieved from GSE54570, which involved expression data of brain tissue from an array of 26 patients with major depressive disorders and controls (18).

### 2.2 Identification of common genes between ED and depression

Gene datasets were filtered and normalized after downloading series matrix files of GSE2457 and GSE54564. After converting the probe name and log2 transformation, the differential expression analysis for the normalized data was performed with the assistance of the “limma” package (21) in R software (version 4.2.1, https://www.r-project.org/) to obtain differentially expressed genes (DEGs) between ED or depression and control groups with the threshold of *p*-value < 0.05. Common DEGs between GSE2457 and GSE54564 were acquired using the intersection function. The results were visualized with a heatmap and Venn diagram by the “pheatmap” package and EVenn (http://www.ehbio.com/test/venn/) (22).

### 2.3 Functional analysis

The functional enrichment analysis could interpret the biological roles of determining genes. Gene Ontology (GO), including biological processes, cellular components, and molecular function, and pathways, including Kyoto Encyclopedia of Genes and Genomes (KEGG) and WikiPathway enrichment analyses, were conducted in the Database for Annotation, Visualization and Integrated Discovery (DAVID) online tool (http://david.ncifcrf.gov). The screening criteria included gene number >2, Ease < 1, and *p*-value < 0.05. The results were visualized by the “circlize”, “ggpubr”, and “ggplot2” packages.

### 2.4 Interactive network and module analysis

Common DEGs between ED and depression were processed in the Search Tool for the Retrieval of Interacting Genes (STRING) database (http://string-db.org) to explore the relationship among proteins of interest. After disregarding disconnected nodes and setting the interaction score > 0.4, a protein–protein interaction (PPI) network was reintroduced in Cytoscape (version 3.7.1, https://cytoscape.org/) for rearrangement. The ClueGO plugin in Cytoscape was used to demonstrate the internal interconnection of ontologies and pathways involved in common DEGs with medium network specificity and showing pathways with *p*-value < 0.05 (23).

In the rearranging network, the Molecular Complex Detection (MCODE) plugin was utilized to conduct module analysis for representing specific molecular complexes with the threshold of degree cutoff = 2 and node score cutoff = 0.2 (24). To present the relationship and select enriched terms, the Metascape (http://metascape.org) online tool was employed with a threshold of *p*-value < 0.05 and enrichment factor >1.5 (25).

### 2.5 Significant shared gene detection and functional interaction

Since dense interaction existed in the present large-scale network, the CytoHubba plugin in Cytoscape was applied to detect significant shared genes based on topological algorithms consisting of BottleNeck, Cluster coefficient, EPC, MCC, and MNC (26). After selecting five ranking methods, the GeneMANIA online tool (https://genemania.org/) was employed to decipher the information on co-localization, co-expression, and functional characterization among these genes (27).

### 2.6 Transcriptional and post-transcriptional analysis

Considering the underlying regulations between transcription factor (TF), miRNA, and genes, significant shared gene-related TFs and miRNAs analysis was implemented in the NetworkAnalyst online tool (https://www.networkanalyst.ca/) based on the RegNetwork repository (28). Then, the coregulatory interactions were visualized in Cytoscape for an optimal layout.

### 2.7 External validation of candidate hub crosslinks

To enhance authority and stringency, the significant shared genes were validated in ED- and depression-related datasets. In GSE206528, after merging and filtering low-expression data, the transcriptome was normalized by NormalizeData, and FindAllMarkers was utilized to analyze featured genes between different groups. These processes were conducted by the “Seurat” package. In GSE54570, DEGs were obtained in similar steps to GSE54564 by the “limma” package. *p*-value < 0.05 was regarded as statistically significant. Furthermore, expression patterns of the significant shared genes were validated in these gene sets to obtain hub crosslinks between ED and depression.

### 2.8 Experimental validation of candidate hub crosslinks

To experimentally validate the candidate hub crosslinks between ED and depression, the corpus cavernosum samples were harvested from control and ED rats. For ED rats, 8-week-old Sprague-Dawley rats were injected with streptozotocin (60 mg/kg) intraperitoneally. Then, rats with fasting blood glucose levels greater than 16.7 mmol/L 3 and 7 days after the injection were considered to be diabetic. After the administration for 10 weeks, an apomorphine test was conducted to assess erectile function based on our previous study (29), and those rats with negative results were treated as ED rats. Electrostimulation was used later to evaluate ED and control rats’ erectile function (29). The study was approved by the Experimental Animal Administration Committee of Wuhan Servicebio Biotechnology in China.

Quantitative real-time PCR was conducted to determine the mRNA levels of control and ED rats. Based on the corresponding protocols, total RNA from six control and ED rats was extracted using the RNA-easyTM Isolation Reagent (G3013, Servicebio, China). Then, cDNA was synthesized from the RNA samples by the Servicebio^®^RT First Strand cDNA Synthesis Kit (G3330, Servicebio, China). Quantitative real-time PCR was operated based on 2×SYBR Green qPCR Master Mix (G3320, Servicebio, China). Detailed information on primer sequences is shown in **Supplementary Table 1**. The mRNA levels of genes were presented as mean ± standard deviation. The comparisons were performed by Student’s *t*-test and shown in the GraphPad software (LLC, San Diego, California, USA).

### 2.9 Subpopulation distribution

The Male Health Atlas database (MHA, http://www.malehealthatlas.cn/) (20) was used to excavate the subpopulation distribution of hub genes in cell clusters of the corpus cavernosum. The database of Human Protein Atlas was used to analyze hub gene localization in brain tissue (HPA, https://www.proteinatlas.org/).

## 3 Results

### 3.1 Identification of common genes between ED and depression

**Figure 1:**
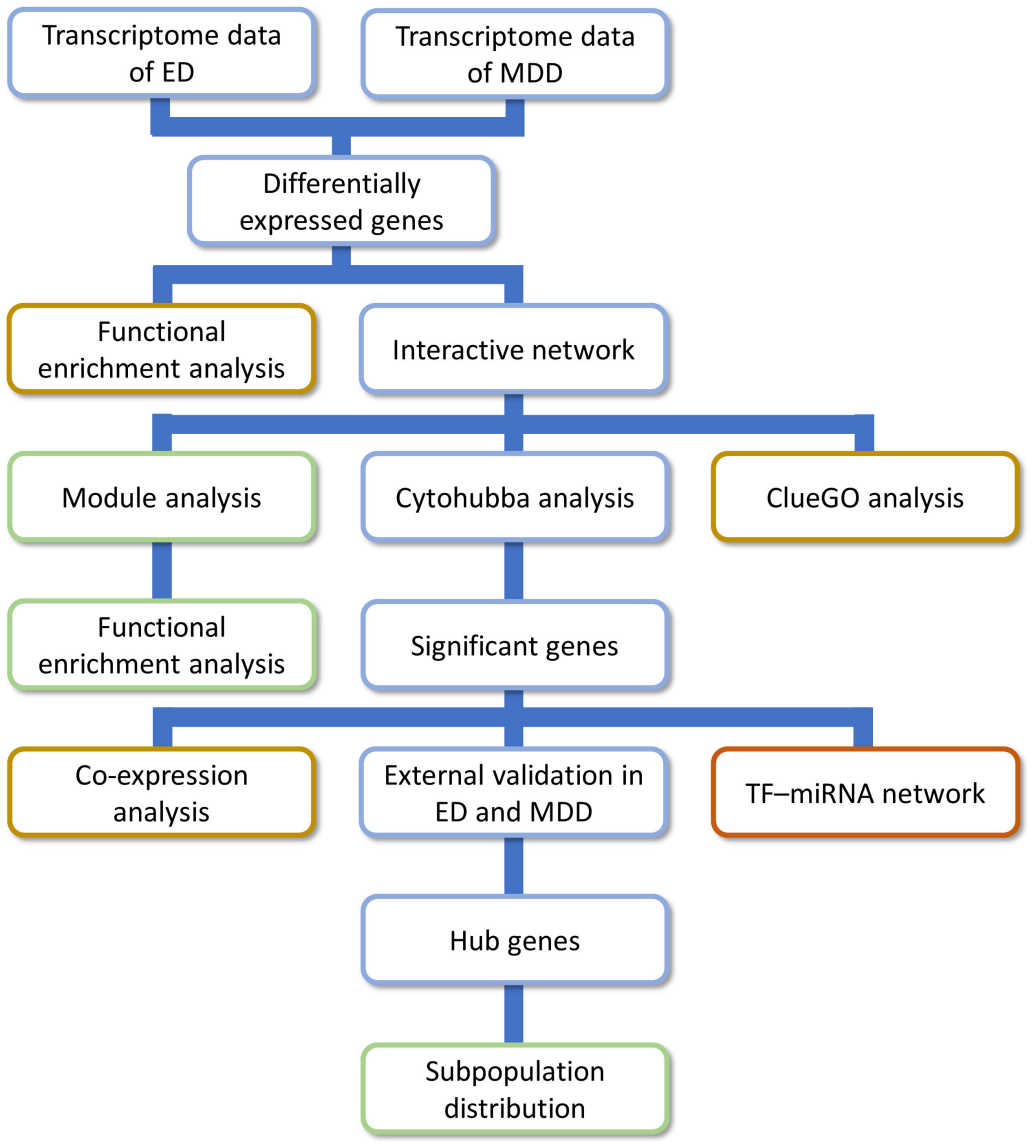
The research approach of the present study. ED, erectile dysfunction; MDD, major depressive disorder.

**Figure 2:**
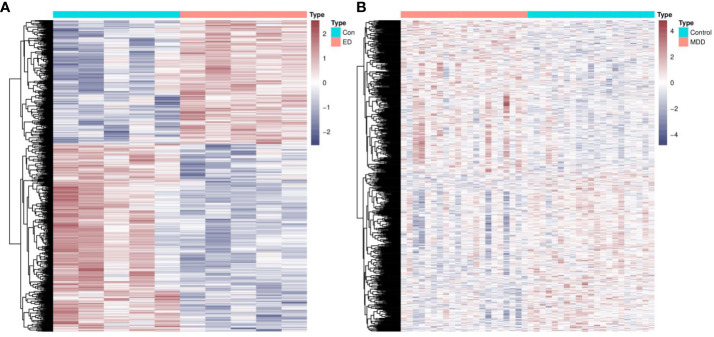
Identification of common genes between ED and depression. **(A)** The heatmap of DEGs between ED and control groups. **(B)** The heatmap of DEGs between depression and control groups. ED, erectile dysfunction; DEGs, differentially expressed genes.

The research approach of the present study is shown in **Figure 1**. Differential expression analysis in GSE2457 revealed a total of 1,570 DEGs between the ED and control groups (**Figure 2A**). Similarly, a total of 1,587 DEGs were excavated between the depression and control groups (**Figure 2B**). Common genes between ED and depression were obtained by the intersection of these two DEGs. Finally, 85 genes were identified for further analysis.

### 3.2 Functional enrichment analysis of common genes

**Figure 3:**
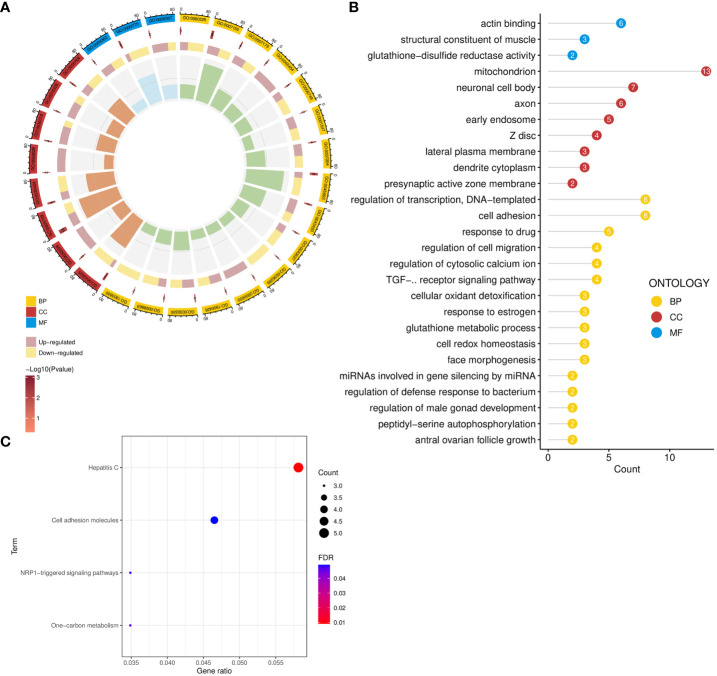
Functional enrichment analysis of common genes. **(A, B)** GO enrichment analysis of common genes. **(C)** Pathway enrichment analysis of common genes. GO, Gene Ontology; BP, biological process; CC, cellular component; MF, molecular function.

GO analysis revealed the biological functions of common genes between ED and depression (**Figure 3A**). Biological processes enriched cellular adhesion, redox homeostasis, glutathione metabolism, and oxidant detoxification. The neuronal cell body, dendrite cytoplasm, axon, and lateral plasma membrane were also enriched in cellular component significantly. Furthermore, these genes were associated with glutathione-disulfide reductase activity, actin binding, and structural constituent of muscle in molecular function (**Figure 3B**). In pathway enrichment analysis, shared pathways contained cell adhesion molecules, one-carbon metabolism, and NRP1-triggered signaling pathways in KEGG and WikiPathway (**Figure 3C**).

### 3.3 Construction of an interactive network and module analysis

**Figure 4:**
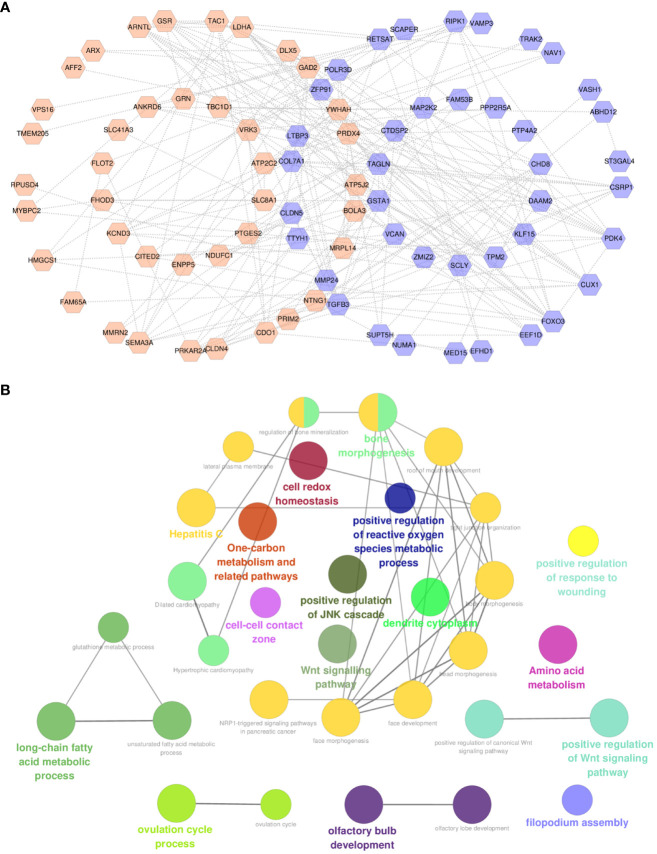
Construction of interactive network and functional analysis. **(A)** Protein–protein interactive network of common genes. **(B)** ClueGO functional analysis of common genes. For **(A)**, orange labels represent upregulated CPRGs and purple labels denote downregulated genes.

A total of 80 protein nodes and 216 interactions constitute an interactive network (**Figure 4A**). In addition to the functions associated with GO and pathway enrichment analysis, these genes also took part in the regulation of reactive oxygen species metabolism, JNK cascade, cell–cell contact zone, and Wnt signaling pathway. Furthermore, long-chain fatty acid and amino acid metabolic processes were observed in the internal interconnection of ontologies and pathways (**Figure 4B**).

**Figure 5:**
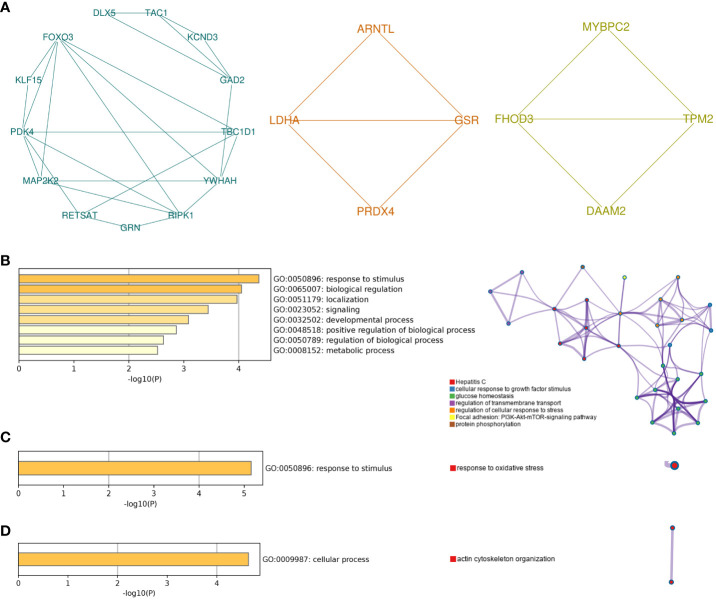
Module analysis of common genes. **(A)** The top three gene clusters based on module analysis. GO biological process and pathway and process enrichment analysis of cluster 1 **(B)**, cluster 2 **(C)**, and cluster 3 **(D)**. For pathway and process enrichment analysis in **(B–D)**, each node represents an enriched term and is colored by cluster ID. GO, Gene Ontology.

Different gene clusters were mined based on functional annotation modules in the following steps. A total of three gene clusters were identified (**Figure 5A**). There were 13 nodes and 24 interactions in cluster 1, focusing on biological regulation, including growth factor stimulus, cellular response to stress, glucose homeostasis, and the PI3K-Akt-mTOR signaling pathway (**Figure 5B**). Four genes in cluster 2 were associated with response to stimulus and oxidative stress (**Figure 5C**). In addition, the cellular process and actin cytoskeleton organization were notable in cluster 3 (**Figure 5D**).

### 3.4 Analysis of the comprehensive proteomic signature of significant shared genes

**Figure 6:**
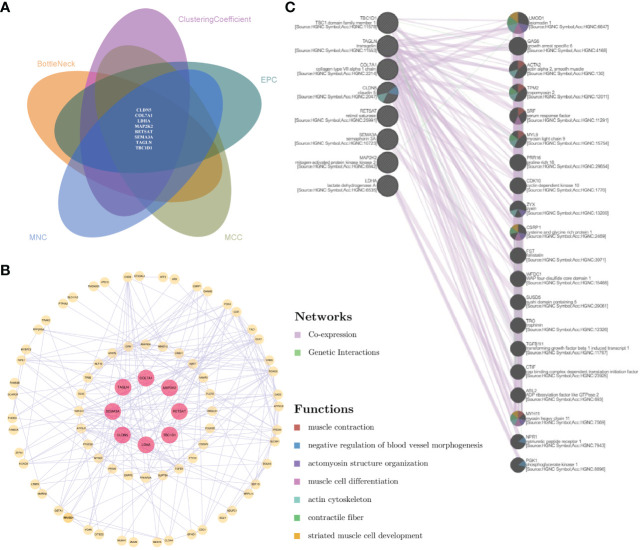
Analysis of the comprehensive proteomic signature of significant shared genes. **(A)** Comparative analysis via topological algorithms in Cytohubba. **(B)** Protein–protein interactive network of significant shared genes. **(C)** The functional interaction in GeneMANIA. For **(C)**, genes on the right side showed the 20 most frequently changed neighboring genes.

To further narrow the gene information and maximize the roles of important genes, comparative analysis based on topological algorithms was conducted, and a total of eight significant shared genes were obtained, namely, CLDN5, COL7A1, LDHA, MAP2K2, RETSAT, SEMA3A, TAGLN, and TBC1D1 (**Figure 6A**). The interaction between significant shared genes and other genes in the protein–protein network is shown in **Figure 6B**. Furthermore, based on co-expression and genetic interactions, GeneMANIA indicated that these genes had a close relationship with blood vessel morphogenesis, muscle cell activity, and actin cytoskeleton (**Figure 6C**). Their biological functions and roles in relevant diseases are listed in **Supplementary Table 2**.

### 3.5 Determination of the transcriptional and post-transcriptional signature

**Figure 7:**
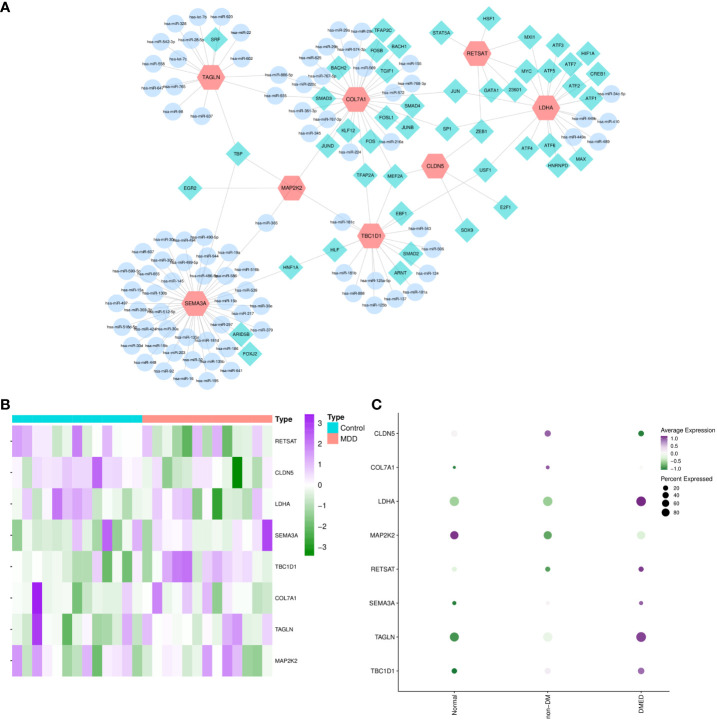
TF–gene–miRNA network and external validation of candidate hub crosslinks. MiRNA–CPRGs network construction and functional enrichment analysis. **(A)** TF–gene–miRNA co-regulatory network. The profiling of significant shared genes in validated depression **(B)** and ED-related gene sets **(C)**. MDD, major depressive disorder; ED, erectile dysfunction; DM, diabetes mellitus; DMED, diabetes mellitus-related erectile dysfunction.

In functional enrichment analysis, miRNA activity was found to be significant in biological processes. Therefore, transcriptional, and post-transcriptional interactions may play an important role in modulating the shared transcriptome. The TF–gene–miRNA network showed 47 TFs and 88 miRNAs (**Figure 7A**). SEMA3A possessed the most miRNA interactions, and most TFs focused on LDHA. Moreover, TBP and MEF2A were common transcription factors involved in three shared genes, which may play a significant role in ED and depression.

### 3.6 External validation of candidate hub crosslinks

After screening and functional analysis step by step, significant shared genes were validated in ED- and depression-related datasets simultaneously to obtain hub crosslinks. After performing differential expression analysis in GSE206528 and GSE54570, respectively, overlapped genes in the two conditions were compared with shared genes (**Figures 7B,C**). It was validated and determined that CLDN5 and TBC1D1 were the hubs of genetic links between ED and depression. Later, the expression profiles of CLDN5 and TBC1D1 were validated in ED rats and controls by quantitative real-time PCR. In **Supplementary Figure 1**, the results of quantitative real-time PCR suggest that notable decreases and increases were observed in the expressions of CLDN5 and TBC1D1 in the ED group compared with the control group (*p*-value < 0.05), emphasizing the critical genetic links of CLDN5 and TBC1D1 between ED and depression.

### 3.7 Subpopulation distribution of hub genes between ED and depression

**Figure 8:**
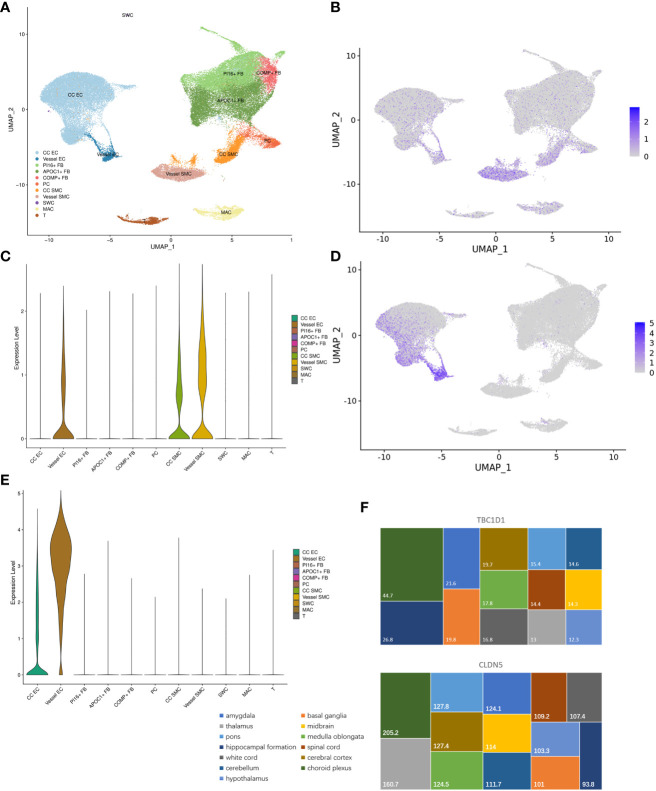
Subpopulation distribution of hub genes between ED and depression. **(A)** The whole cellular distribution in human corpus cavernosum. **(B, C)** Expression distribution of TBC1D1 in human corpus cavernosum. **(D, E)** Expression distribution of CLDN5 in human corpus cavernosum. **(F)** Expression distribution of TBC1D1 and CLDN5 in brain tissue. ED, erectile dysfunction.

Subpopulation distribution in specific tissues is vital for performing the functions of genes. In MHA, the human corpus cavernosum included 11 types of cells (**Figure 8A**). TBC1D1 was predominantly localized in vessel endothelial cells, corpus cavernosum, and vessel smooth muscle cells (**Figures 8B,C**). CLDN5 was found to be enriched in the corpus cavernosum and vessel endothelial cells (**Figures 8D, E**). In brain tissue, TBC1D1 had a higher distribution in choroid plexus and hippocampal formation. CLDN5 targeted the choroid plexus, thalamus, pons, and cerebral cortex (**Figure 8F**).

## 4 Discussion

The presence of both ED and depression significantly impacts the psychosocial wellbeing and quality of life of couples (30). Although high comorbidity between the two conditions is apparent, there remains considerable uncertainty around the pathogenetic nature. There is no clinical, epidemiological association between ED and depression that is sufficient to explain the co-occurrence of the diseases. In this study, we comprehensively analyzed the shared transcriptome between ED and depression using multi-omics methods for the first time. Hub crosslinked genes and correlative signaling pathways were identified and validated based on multi-dimension data.

Considering the heterogeneity in ED and depression-related tissues, the differential expression and comparative analysis identified only 85 common genes. Functional enrichment analysis revealed that these genes were involved in cell redox homeostasis, glutathione metabolic process, glutathione-disulfide reductase activity, and cellular oxidant activities. The results indicate that oxidative stress is crucial in ED and depression. Cumulative reactive oxygen species during ED inhibit the synthesis and bioavailability of NO, which is an important mediator of endothelium-dependent vasodilation in the corpus cavernosum (31). NADPH oxidases are the main source of endogenous reactive oxygen species, which have been noted in ED tissues (29). The use of antioxidants has shown promising results in the treatment of ED. Owing to high oxygen consumption and weak defenses, the brain is more susceptible to oxidative injury, and oxidative stress is believed to be the main cause of major depressive disorder (32). The activity of glutathione peroxidases is lowered and contributes to reactive oxygen species in patients with depression (33). The maintenance of cellular redox homeostasis was crucial in ED and depression, indicating the important shared mechanism.

Since neurologic disorders may play a significant role in linking ED and depression, cellular component analysis was expected to enrich neuronal cell body, dendrite cytoplasm, and axon. Pathway enrichment analysis revealed changes in NRP1-triggered signaling pathways and cell adhesion molecules. In a model of mouse depression, NRP1 was found to be involved in hippocampal neurogenesis and neuroplasticity via miR-30 (34). Neuronal cell adhesion could also be considered a potential marker in antidepressant response (35). Dysfunction in endothelial cell-to-cell junctions was found to be significant in the pathogenesis of hypercholesterolemia-induced ED (36). Subsequent functional enrichment in gene clusters focused on the reactive oxygen species metabolic process, the cell–cell contact zone, and the nutrient metabolic process. It is important to consider these factors in relation to the shared association between ED and depression in future studies.

After a comprehensive analysis of the proteomic signature, a total of eight significant shared genes, namely, CLDN5, COL7A1, LDHA, MAP2K2, RETSAT, SEMA3A, TAGLN, and TBC1D1, were identified. COL7A1 encodes the alpha chain of type VII collagen. Available research concentrated on COL7A1 in all forms of dystrophic epidermolysis bullosa (37, 38). As an important component of the tumor microenvironment, COL7A1 demonstrated prognostic value in patients with gastric cancer and pancreatic cancer (39, 40). Further study of COL7A1 was warranted due to its promising function. LDHA belongs to the lactate dehydrogenase family. It is associated with pyruvate metabolism, glycolysis, and oxidoreductase activity (41). Significant change in LDHA was observed in cerebral gluconeogenesis in chronic stress-related depression (42). Gong et al. (43) reported that Angelicae Sinensis Radix modulated energy metabolism in depression by inhibiting the expression of LDHA. Since oxidoreductase activity was predominant in the process of ED, it was plausible to explore LDHA in the corpus cavernosum. MAP2K2 plays a critical role in mitogen growth factor signal transduction, which is enriched in the central nervous system. It is lower in individuals with psychiatric disorders (44). Similarly, it is also involved in the process of ED (45). RETSAT, known as retinol saturase, is upregulated in ED and diabetic rats (46). It is also a potent modulator of the cellular response to oxidative stress and the generation of reactive oxygen species *in vivo* and *in vitro* (47). SEMA3A is a member of the semaphorin family with an Ig-like C2-type domain and is involved in axon guidance and neuronal connectivity. Existing evidence indicates that SEMA3A alleles are associated with genetic disorders in the central nervous system, including autism spectrum disorders and neuronal migration (48). Zhou et al. (49) demonstrated that rs139438618 at the SEMA3A locus is significantly associated with the comorbidity of alcohol dependence and major depressive disorder. Whether SEMA3A is related to ED is unknown, and this may be explored in the future. TAGLN is ubiquitously expressed in vascular and visceral smooth muscle as a marker of smooth muscle differentiation. In a model of myostatin homozygous mutant knockout pig, increased expression of TAGLN was noted in the penile corpus cavernosum, which could be a target for treating ED (50). Moreover, angiogenesis may be a possible link between TAGLN and depression (51–53).

After identification and validation, CLDN5 and TBC1D1 were regarded as the hub genetic links of ED and depression. CLDN5, also known as Claudin 5, belongs to endothelium-specific cellular junction proteins. Its decrease could reduce endothelial content in the corpus cavernosum and deteriorate ED (36). Furthermore, its family may serve as an endothelial barrier to reflect hemodynamic changes in the corpus cavernosum (54). On the other hand, CLDN5 was associated with blood–brain barrier permeability. Menard et al. (55) found that social stress would inhibit the expression of CLDN5, destroy blood–brain barrier integrity, and induce depression ultimately (56). Furthermore, TNF-α could affect blood–brain barrier permeability and CLDN5 expression of endothelial cells in major depressive episodes (57). Stress-related brain entry of inflammatory molecules and IL-6–CLDN5 interaction are also involved in the development of depression (58). TBC1D1 plays a role in regulating cell growth and differentiation. In the available studies, TBC1D1 was not reported to be involved in the pathogenesis of ED or depression, but it is one of the suicide attempt polygenes that may have a link to depression (59). Therefore, it is significant to explore the specific mechanisms of CLDN5 and TBC1D1 in the crosslink of ED and depression.

Some limitations were found in this study. Although comprehensive excavation and validation were conducted based on tissue and human samples, biases could exist due to limited sample size, diabetic state, and heterogeneity across different species. A total of 85 genes generated from comparative analysis lacked appropriate statistical correction. Furthermore, these findings could not establish a causal relationship between the shared transcriptomic profiles of the identified genes and such disorders. Further prospective studies are needed. Moreover, psychosocial factors play an important role in the progress of depression. Chiu et al. (60) reported that social trauma and dissociation contributed to psychotic symptoms in patients with major depressive disorder. The psychosocial contents were insufficient due to the genetic basis of this research. The molecular mechanisms underlying the shared pathogenesis in ED and depression have not been explored enough, and we will investigate these mechanisms in future research.

## 5 Conclusions

Our multi-omics analysis revealed the shared biological roles and transcriptomic profiles of ED and depression for the first time. After identification and validation, CLDN5 and TBC1D1 were selected as the hub genetic links between the two conditions. These results provided referential molecular mechanisms in concurrent depression and ED.

## Data availability statement

The original contributions presented in the study are included in the article/**Supplementary Files**, further inquiries can be directed to the corresponding author/s.

## Author contributions

Conceptualization: PY, YC, and QM; Formal analysis: PY, TS, LC, and YW; Writing-original draft: PY; Funding acquisition: PY and YC; Data collection and analysis: PY, TS, and TL; Review & editing: PY, YC, TL, and QM. All authors contributed to the article and approved the submitted version.
