# Supplementary material for: Exploring potential genes and mechanisms linking erectile dysfunction and depression
Source: Front Endocrinol (Lausanne). 2023 Dec 4;14:1221043. doi: 10.3389/fendo.2023.1221043 (PMC10726033; doi:10.3389/fendo.2023.1221043)
Supplement: Supplementary file 1 [file DataSheet_1.docx]

Supplementary Material

**Supplementary Table 1**

The oligonucleotide sequences of primers used in quantitative real-time PCR.

| Gene | Primer type | Sequence/Target sequence |
| --- | --- | --- |
| CLDN5 | Forward | 5’-GGCACTCTTTGTTACCTTGACC-3’ |
|  | Reverse | 5’-CCAGCTCGTACTTCTGAGACACC-3’ |
| TBC1D1 | Forward | 5’-GAGGCAATCACATTCACAGCG-3’ |
|  | Reverse | 5’-CAAAGGCGGACTTGCTTCATC-3’ |
| β-actin | Forward | 5’- GGAGCGAGATCCCTCCAAAAT-3’ |
|  | Reverse | 5’- GGCTGTTGTCATACTTCTCATGG-3’ |

**Supplementary Figure 1**

The mRNA levels of CLDN5 and TBC1D1 between ED and control groups.


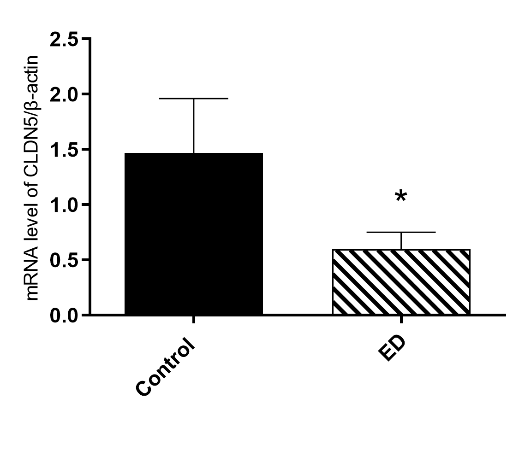

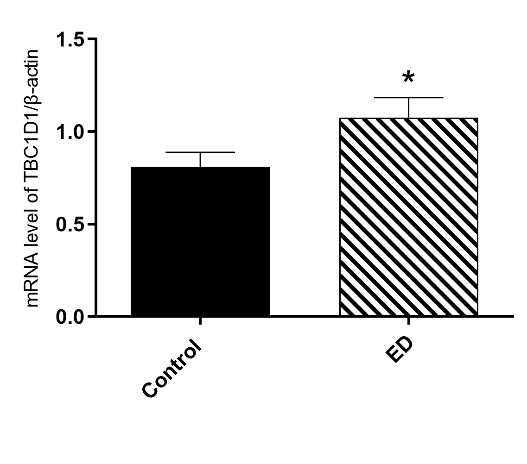


A B

The mRNA levels of CLDN5 (A) and TBC1D1 (B) between ED and control groups. * indicates P < 0.05.

**Supplementary Table 2**

Reported roles of the shared genes identified by us in the relevant diseases.

| **Gene** | **Gene full name** | **Reported roles of gene in the relevant diseases** | **References** |
| --- | --- | --- | --- |
| **CLDN5** | Claudin 5 | Its decrease could reduce endothelial content in the corpus cavernosum and deteriorate ED. CLDN5 could function as a barrier in endothelial cells, reflecting hemodynamic changes in the corpus cavernosum. Also, it had a relationship with blood-brain barrier permeability. Social stress would inhibit the expression of CLDN5, destroy blood-brain barrier integrity, and induce depression. | PMID:  19453913  19622796  29184215  31974313 |
| **COL7A1** | Collagen Type VII Alpha 1 Chain | COL7A1 was involved in all forms of dystrophic epidermolysis bullosa. It was an important component of the tumor microenvironment, and showed prognostic value in patients with gastric cancer and pancreatic cancer. | PMID:  28549954  33914976  34512204  32355831 |
| **LDHA** | Lactate Dehydrogenase A | LDHA was associated with pyruvate metabolism, glycolysis and oxidoreductase activity. Its expressed level changed in cerebral gluconeogenesis in chronic stress-related depression significantly. Angelicae Sinensis Radix modulated energy metabolism in depression through the inhibition of the expression of LDHA. | PMID:  34404767  35448030  30894817 |
| **MAP2K2** | Mitogen-Activated Protein Kinase Kinase 2 | MAP2K2 was enriched in the central nervous system and played a critical role in mitogen growth factor signal transduction. It was lower in individuals with psychiatric disorders. It was also involved in the process of ED. | PMID:  19913919  23889981 |
| **RETSAT** | Retinol Saturase | The expressed level of RETSAT was up-regulated in ED and diabetic rats. It was a potent modulator of the cellular response to oxidative stress and the generation of reactive oxygen species in vivo and in vitro. | PMID:  34933709  28927883 |
| **SEMA3A** | Semaphorin 3A | SEMA3A was involved in axon guidance and neuronal connectivity. Its alleles were associated with genetic disorders in the central nervous system including autism spectrum disorders and neuronal migration. Also, rs139438618 at SEMA3A locus was significantly associated with the comorbidity of alcohol dependence and major depressive disorder. | 25769423  29071344 |
| **TAGLN** | Transgelin | TAGLN was expressed in vascular and visceral smooth muscle as a marker of smooth muscle differentiation. Increased expression of TAGLN was noted in the penile corpus cavernosum. Besides, angiogenesis may be a possible link between TAGLN and depression. | PMID:  36194928  34338296  35618888  24331740 |
| **TBC1D1** | TBC1 Domain Family Member 1 | TBC1D1 had a role in regulating cell growth and differentiation. It was one of the suicide attempt polygenes studied in suicidal behavior, which could be the possible link to depression. | PMID:  26666204 |
